# A Medical Causerie

**Published:** 1915-08-07

**Authors:** 


### A MEDICAL CAUSERIE.

The Scottish War Emergency Committee will doubtless appreciate the compliment involved in the recent decision to organise a similar Committee for England, Ireland, and Wales. Presumably the first duty of the new Committee will be to ascertain, by means of a census, what medical practitioners are available for military service, either at home or abroad. This will be no light task, and probably the most effective method would be by dividing the country into relatively limited areas and getting some local representative medical organisation to act as agents in each of these. Then, if the Scottish precedent is followed, each district will be told what number of medical men it is expected to supply, and here obviously there must be a representative organisation familiar with local conditions and able to exercise personal influence. The time for vague general appeals has passed, and it rests with the profession to see that the needs of the Army are supplied. Unless the new Committee succeeds in doing this it will not have justified its existence. Merely to accumulate figures and statistics is, in the circumstances, a vain proceeding. The Metropolitan Committee, it is understood, reached this point, but it does not seem to have passed it. At least no statement has been published showing the exact number of practitioners who have been led by the Committee to join the Army. The financial difficulty remains, and there is no doubt that numbers of the younger men who are already in practice feel that the terms offered are not adequate to meet the risk of loss which absence from practice necessarily entails. The Treasury, however, is inflexible, and presumably, therefore, the authorities are getting what doctors they need for the moment. How far Sir Alfred Keogh is satisfied no one knows, but it is impossible to believe that he has received all the help for which he asked some months ago. It has been suggested that the profession generally should raise a fund to compensate those who volunteer for active service. Certainly this would be a very impressive thing to do, but one cannot deny the force of the comment that if the country needs the doctors adequate payments should be made to them from public funds.

According to rumour, one West End practitioner, after the Director-General's appeal, was moved to call at the War Office, and when asked what he wanted, replied, "Oh! I don't want anything. I thought it was you who were asking for help, and so I came to see what I could do for you." So literal a reading of the situation was not to the taste of the officials, and the would-be volunteer was urged to take an unceremonious departure.

A good deal of irresponsible criticism is being passed on the War Office for what is called the wasteful distribution of doctors. Practitioners, it is said, are in large numbers being kept at stretcher drill and other routine duties when they are quite fit to undertake active service. On the same principle hostile remarks are made on the arrangements at the new King George Hospital, where some thirty young men are employed as house physicians and house surgeons. But surely some confidence must be placed in the Director-General, who has to be ready to supply a demand for a large number of doctors almost at a moment's notice. The situation at the time being is perhaps not acute, but provision must be made for the near future, when larger armies are in the field and the fighting becomes more severe. The return of the casualties from the Dardanelles is sufficient to explain the claim which was recently made on the Army Medical Department, and everything goes to show that the strain is likely to be increased rather than diminished. Men can hardly be taken from medical practice and placed immediately amidst the complex machinery of the Army; some measure of routine training is essential, for while everybody just now talks of red tape with scorn, a certain amount of it is necessary for smooth working. As for the King George Hospital, a certain number of juniors as residents cannot be avoided in an institution where many serious cases are admitted and treated. This experience, too, is a most valuable training for service with an army in the field. The point for criticism is less the numbers employed than the length of service. If these junior positions can be held indefinitely the arrangement is certainly a bad one. But if there is a limit of, say, six months' service, with entrance into the R.A.M.C. at the end of the term, then the hospital becomes a valuable training school, where men are specially fitted for Army duties and are in due time sent forward for active service.

The appeal of the London School of Medicine for Women for £15,000, in order to build new laboratories, has not escaped comment. In ordinary times the proposal doubtless would have been welcomed, but in existing circumstances the question may well be asked whether the scheme is really urgent. Laboratory accommodation in London, in view of the diminished number of male medical students, must be abundant, and surely by a little organisation all that is required for the time being could be secured, even if the classrooms at Hunter Street are crowded out. What certain distinguished politicians who have allowed their names to be attached to the scheme know about the subject it is difficult to imagine. Methods which are suitable enough in the promotion of a charity bazaar are hardly usual in seeking the endowment of science. And, in any event, there is the supreme national claim that money is needed for the prosecution of the war and for the support of the various funds which, directly or indirectly, minister to this end. Not that £15,000 is a specially large sum; but if one particular interest may put forward an appeal, why not others? Probably everyone will say the example ought not to be followed. But if so, it surely ought not to have been set.

